# Local and Interregional Neurochemical Associations Measured by Magnetic Resonance Spectroscopy for Studying Brain Functions and Psychiatric Disorders

**DOI:** 10.3389/fpsyt.2020.00802

**Published:** 2020-08-11

**Authors:** Jun Shen, Dina Shenkar, Li An, Jyoti Singh Tomar

**Affiliations:** Molecular Imaging Branch, National Institute of Mental Health, Bethesda, MD, United States

**Keywords:** glutamate, GABA, neurochemical correlations, magnetic resonance spectroscopy, spectral editing, psychiatric disorders

## Abstract

Magnetic resonance spectroscopy (MRS) studies have found significant correlations among neurometabolites (*e.g.*, between glutamate and GABA) across individual subjects and altered correlations in neuropsychiatric disorders. In this article, we discuss neurochemical associations among several major neurometabolites which underpin these observations by MRS. We also illustrate the role of spectral editing in eliminating unwanted correlations caused by spectral overlapping. Finally, we describe the prospects of mapping macroscopic neurochemical associations across the brain and characterizing excitation–inhibition balance of neural networks using glutamate- and GABA-editing MRS imaging.

## Introduction

*In vivo* MRS is the only noninvasive technique that can directly measure brain chemicals *in vivo*. Using techniques similar to MRI, MRS can measure concentrations of many neurometabolites as well as metabolic fluxes from localized brain regions (1). Over the past decades, MRS studies have found biochemical abnormalities in essentially all neuropsychiatric disorders, providing important insights into our understanding of etiologies and treatments of various brain diseases. These studies have, in most cases, focused on alterations in the concentrations of individual brain neurometabolites by comparing them among different cohorts and/or effects of treatments.

Many significant correlations among neurometabolites and between individual neurometabolites and non-MRS measures of brain function and disorders have been reported more recently. Altered correlations have been found in neuropsychiatric disorders, revealing abnormal neurochemical associations under pathophysiological conditions [e.g., (2–12)]. The strong correlations among N-acetylaspartate (NAA)/choline in precentral gyrus, midcingulate cortex, and thalamus found in healthy subjects were absent in patients with amyotrophic lateral sclerosis (4). Correlation between hippocampal Glx (glutamate + glutamine) and NAA has been demonstrated to be a more sensitive biomarker differentiating between healthy controls and schizophrenia patients than either neurometabolite alone (6, 7). In patients with subclinical hepatic encephalopathy the occipital lobe phosphodiester measured by ^31^P MRS and Glx levels were found to be negatively correlated (13). Interregional correlations of glutamate and GABA levels have also been reported in many studies [e.g., (8, 14–17)]. These studies have clearly demonstrated that neurochemical associations are abnormally altered in many brain disorders, but the absolute strengths of these correlations measured by different MRS methodologies have been inconsistent or controversial [e.g., (5, 14, 15, 18–23)]. In particular, the effects of spectral overlap on the observed neurometabolite correlations have yet to be illustrated although they can significantly confound the intrinsic neurochemical correlations of interest.

Many studies of neurochemical associations rely on spectral fitting [e.g., (24)] to extract neurometabolite concentrations from overlapping signals. When there is significant spectral overlap between two signals overestimate of one signal is statistically correlated with underestimate of the other signal and *vice versa*, even when there exists no neurochemical correlation between the two signals. In addition, this type of statistical correlations can propagate due to the intensity constraints imposed by LCModel (24) or overlapping with neurochemically correlated signals. When neurometabolite concentrations are correlated with other measurements (e.g., behavior, resting state fMRI functional connectivity, or gene expression), statistical correlations due to spectral overlap among MRS measurements can also affect correlations between MRS measurements and non-MRS measurements.

In this article we review dominant metabolic pathways connecting major neurometabolites (25–27) which underpin the neurochemical associations detected by MRS correlation studies. Non-MRS neurochemical studies of animal models that found correlated changes in neurometabolite concentrations under various pathophysiological conditions are also discussed. Monte Carlo simulations are performed to demonstrate the existence of statistical correlations that originated from spectral overlap. Finally, we discuss MRS techniques that eliminate spectral overlap and associated statistical correlations. We hope that these discussions will spur interest in developing MRS techniques for mapping neurochemical associations across the brain to facilitate a variety of clinical investigations. In particular, since glutamate and GABA play dominant roles in the excitation–inhibition balance (28, 29) and in many neuropsychiatric disorders (30–33), MRS characterization of glutamate–GABA associations among the nodes of neural networks may provide considerable insight into the interactions between glutamatergic and GABAergic systems and their abnormalities.

## Metabolic Pathways Underlying Neurochemical Associations

Predominant metabolic pathways connecting NAA, glutamate, glutamine, and GABA are reviewed in detail. For clarity, a table summarizing these pathways is provided (**Table 1**).

**Table 1:** Predominant metabolic pathways of NAA, glutamate, glutamine and GABA in brain.

Neurometabolites	Anabolic enzymes	Catabolic enzymes
NAA	NAA synthase	aspartoacylase
glutamate	aspartate aminotransferase, glutaminase, glutamate dehydrogenase	aspartate aminotransferase, glutamine synthetase, glutamate dehydrogenase, glutamic acid decarboxylase
glutamine	glutamine synthetase	glutaminase
GABA	glutamic acid decarboxylase	GABA transaminase

### NAA-Glutamate Association

NAA is the most abundant free amino acid derivative in the CNS. NAA is found almost exclusively within the nervous system. In the adult brain, it is mostly confined to neurons. As such, it is of great clinical interest as it has been considered as a neuronal marker for assessment of neuronal viability in a variety of neuropsychiatric disorders using proton MRS. For example, NAA level is markedly reduced within the infarct of stroke patients (34), while a higher NAA level is associated with a better clinical outcome (35). Despite the intense interest in NAA, both its physiological and metabolic roles in normal brain functions as well as in neuropsychiatric disorders remain poorly understood [for a review of the putative role of NAA, see (36)].

Important metabolic associations exist among major neurometabolites observable by MRS through precursor–product relationships and sharing common substrates (26, 37, 38). Glutamate, the most abundant intracellular amino acids in mammals, is a key component of intermediary metabolism and a precursor of numerous cellular components including proteins as well as neurometabolites such as GABA, N-acetylaspartylglutamate (NAAG), and glutathione (26). As glutamate is also the primary excitatory neurotransmitter in the CNS, it is not surprising that the proton MRS has found abnormal glutamate levels in many neuropsychiatric disorders including multiple sclerosis (39), major depression (40), and bipolar disorder (41) where glutamatergic dysfunction is broadly implicated.

Glutamate is primarily synthesized by transamination from *α*-ketoglutarate catalyzed by aspartate aminotransferase (25):

glutamate+oxaloacetate↔α-ketoglutarate + aspartate

Glutamate also is produced, to a much lesser extent, from *α*-ketoglutarate and ammonium *via* glutamate dehydrogenase, from glutamine *via* hydrolysis catalyzed by phosphate-activated glutaminase, by other transamination reactions that use *α*-ketoglutarate as receptor of the amino group, and during protein turnover (42).

The transaminases of importance for maintenance of glutamate homeostasis in the brain are mainly aspartate aminotransferase, branched-chain aminotransferase, and alanine aminotransferase with aspartate aminotransferase dominating overwhelmingly, representing >97% of the glutamate-related aminotransferase activities (26). ^13^C magnetization transfer MRS experiments have shown that the aspartate aminotransferase reaction is extremely fast in the brain *in vivo* (43). This rapid transamination by aspartate aminotransferase predominates in the formation of glutamate in the CNS, forming strong metabolic coupling between glutamate and aspartate (25). The tight connection between glutamate and aspartate becomes conspicuous under many pathophysiological conditions where the brain is challenged or perturbed metabolically. For example, during hypoglycemia a decrease in glutamate concentration was accompanied by an increase in aspartate concentration (44–46). Similarly, barbiturate anesthesia and hypothermia were also found to lower the concentration of *α*-ketoglutarate accompanied by reduced glutamate concentration and increased aspartate concentration (47–49). These correlated changes in glutamate and aspartate were explained by a sizable shift in the aspartate aminotransferase reaction towards aspartate formation at the cost of a reduction in glutamate concentration (25, 26). In contrast, both hypocapnia and hypoxic hypoxia are associated with an increase in glutamate concentration and a reduction in aspartate concentration with the aspartate aminotransferase reaction shifting in the opposite direction (25, 26, 50).

A strong metabolic coupling between glutamate and aspartate mediated by the rapid and ubiquitous aspartate aminotransferase reaction also affects NAA, the dominant signal in proton MRS, as NAA is primarily synthesized from acetyl coenzyme A (CoA) and aspartate by NAA synthase (51):

$$\text{acetyl}-\text{CoA}+\text{aspartate}\to \text{CoA}+{\text{H}}_{}^{+}+\text{NAA}$$

In addition to this indirect connection from glutamate to NAA synthesis *via* aspartate, both glutamate and NAA are products of NAAG catabolism catalyzed by N-acetylated-α-linked-amino dipeptidase (52–54). The deacetylation of NAA catalyzed by aspartoacylase has also been proposed as a significant metabolic pathway for NAA to act as a reservoir for glutamate in brain (55).

### Glutamate–Glutamine Association

In contrast to glutamate, which is predominantly located in glutamatergic neurons, glutamine is primarily an astrocytic chemical. In the MRS literature glutamate + glutamine is often collectively referred to as Glx as at lower magnetic fields it has been difficult to separate the two spectroscopically. Abnormal glutamine concentrations have been found in several brain disorders including cancer, hepatic encephalopathy, and other neuropsychiatric disorders (56, 57).

**Figure 1:**
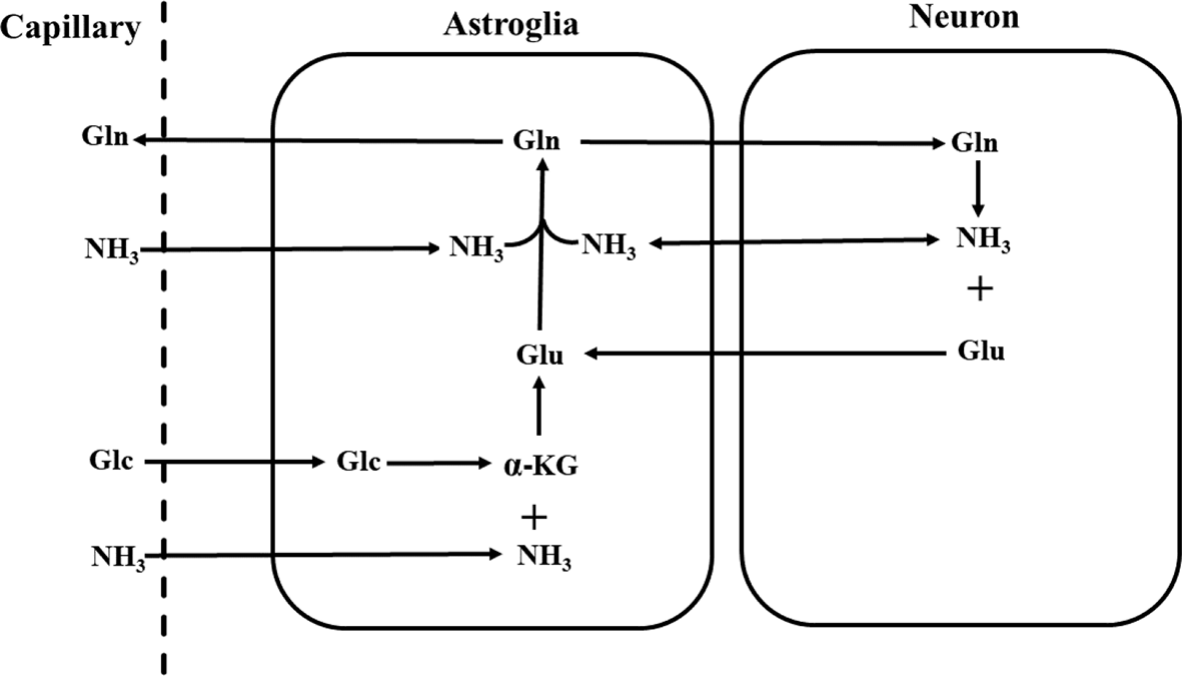
Schematic diagram of the glutamate–glutamine neurotransmitter cycle between neurons and astroglia (58). Glutamate (Glu) is taken up from the synaptic cleft into astroglia. There glutamate is converted to glutamine (Gln) by glutamine synthetase. The inactive glutamine is released by the astroglia, enters the neurons, and then is converted into glutamate by phosphate-activated glutaminase. Glc, glucose; *α*-KG, *α*-ketoglutarate; NH_3_, ammonia.

Although the overall glutamate pool in neural tissues rapidly turns over fueled by primarily glucose under normal physiological conditions, glutamate released from nerve terminals is replenished by astroglial glutamine *via* the glutamate–glutamine neurotransmitter cycle [**Figure 1**; (26, 59)]. The negatively charged highly hydrophilic glutamate cannot diffuse across cell membranes. The concentration of glutamate in the extracellular space is extremely low due to its rapid uptake into the astroglia facilitated by high-affinity Na^+^-dependent transport systems against a large concentration gradient (60–62). Once taken up into the astroglial cells, glutamate is converted into glutamine by glutamine synthetase:

$$\text{glutamate}+{\text{NH}}_{3}+\text{adenosin triphosphate }\to \text{glutamine}+\text{adenosine diphosphate}+{\text{P}}_{\text{i}}$$

or oxidized by assimilation into the tricarboxylic acid cycle of astroglial cells (26, 63). Once formed, glutamine readily enters nerve terminals by its own low affinity transport system or by simple diffusion. There the phosphate-activated glutaminase converts it into glutamate (26):

$$\text{glutamine}+{\text{H}}_{2}\text{O}\to \text{glutamate }+{\text{ NH}}_{4}^{+}$$

A large number of neurochemical as well as autoradiographic studies have confirmed that glutamate is selectively taken up by astroglial cells and then converted into glutamine, while glutamine preferentially enters the neurons and is converted into glutamate there (64). *In vivo* ^13^C and ^15^N MRS studies have quantitatively measured the glutamate–glutamine cycling flux in rodent and human brains [e.g., (58, 65–69)]. Results from these studies have demonstrated that the glutamate–glutamine cycle between glutamatergic neurons and astroglia is metabolically significant, providing a major connection between glutamate and glutamine in the brain (70). In addition, over the range of glutamate concentrations found in the nerve terminals, product inhibition appears to be the main mechanism of control of glutaminase activity with glutamate significantly attenuating the activity of glutaminase (71).

The metabolic connection between glutamate and glutamine is manifested in many brain disorders and animal models. For example, elevated ammonia level in the brain is associated with increased glutamine synthesis for ammonia detoxification (67). It has been reported that in hyperammonemia and hepatic encephalopathy the elevation of glutamine level in the brain is accompanied by a reduced glutamate level as glutamate acts as a receptor for the excessive ammonia (72). The changes in glutamate and glutamine levels, however, do not necessarily go in opposite directions. Both glutamate and glutamine are abundant in the brain. Despite efforts to understand the roles of glutamate and glutamine, the reason for maintaining relatively high concentrations of glutamate and glutamine in the brain is still poorly understood. It is possible that a high concentration of glutamate and glutamine facilitates the generally high metabolic activities in the brain because glutamate and glutamine are key components of intermediary metabolism (25). They are also precursors of many other cellular components (26). Their role as “energy reservoirs” is particularly clear when the brain is under metabolic stress. For example, when glucose is scarce, such as in hypoglycemia, both glutamate and glutamine act as energy fuels. As a result, the concentrations of both glutamate and glutamine are reduced in synchrony during hypoglycemia and in many other pathophysiological conditions when normal oxidative metabolism is impaired (45, 73).

### Glutamate–GABA Association

While glutamate is the major excitatory neurotransmitter in the mammalian brain, GABA is the major inhibitory neurotransmitter. Since the initial detection of reduced GABA levels in epilepsy patients by MRS (74) and a strong correlation between GABA levels and seizure control in epilepsy patients treated by vigabatrin (75), MRS of GABA has greatly advanced both in terms of MRS methodologies and their clinical applications in studying GABAergic abnormalities in neuropsychiatric disorders.

Like glutamate, GABA metabolism proceeds through important intermediates of the tricarboxylic acid cycle. When GABA was first discovered (76) it was realized that GABA was formed from glutamate. Later studies identified that the principal pathway of GABA goes through *α*-decarboxylation of glutamate *via* glutamic acid decarboxylase which converts glutamate directly into GABA (77):

$$\text{glutamate}\to \text{GABA }+{\text{ CO}}_{2}$$

The source of the GABA precursor is believed to be dominated by neuronal glucose with astroglial glutamine playing a smaller role (78–80). GABA synthesis from putrescine and other polyamines is metabolically insignificant in the brain although polyamines play an important role in the developing brain (81).

A fundamental aspect of glutamate–GABA association is the excitation–inhibition balance in the brain (32, 82) because of their roles as the dominant excitatory and inhibitory neurotransmitters, respectively, in the CNS. Glutamatergic neurons (e.g., cortical pyramidal neurons) receive a significant degree of GABA_A_-mediated inhibition through interneurons (83). Balanced excitation and inhibition facilitate normal brain functions, and failure to maintain excitation–inhibition balance underlies dysfunction in many brain disorders (32, 33, 84). Many studies have also revealed altered glutamate and GABA. For example, GABA levels were found to decrease, whereas Glx levels increased with increasing visual input in the occipital cortex of healthy subjects (85). Both glutamate and GABA increased following vigorous exercise (86). In autistic patients the frontal lobe [GABA]/[Glu] ratio was found to be significantly lower, suggesting abnormality in the regulation between GABA and glutamate (87). Abnormalities in MRS measures of glutamate and/or GABA in many other neuropsychiatric disorders have also been reported and reviewed [e.g., (40, 88–90)].

Long range excitatory and inhibitory interactions between functionally connected brain regions are well established (91–93). The strong coupling between glutamatergic neurotransmission and total GABA level is supported by a large body of *in vitro* and *in vivo* evidence [e.g., (16, 75, 94–100)]. A large number of neuroimaging studies have also shown that total glutamate or Glx concentration is significantly correlated with neural activity or glutamatergic neurotransmission [e.g., (28, 101–103)]. Correlations of total glutamate and total GABA levels locally and among regions functionally connected in a specific neural network have also been reported [e.g., (15–17, 19)].

## Statistical Correlations Among MRS Signals Due to Spectral Overlap

**Figure 2:**
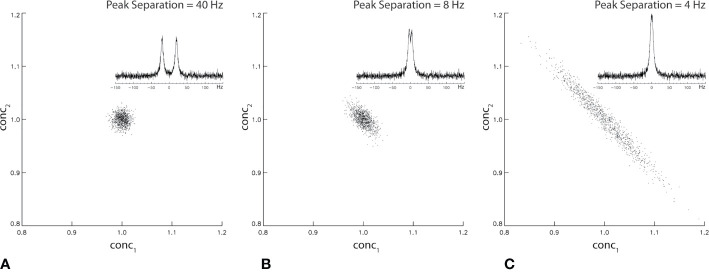
Monte Carlo simulation of statistical correlation between two unrelated Lorentzian singlets with signal-to-noise ratio = 20. The half-height linewidth of the two peaks is 8 Hz. The same spectral fitting process was repeated 1,000 times with the same noise level but different noise realizations. For 40 Hz **(A)**, 8 Hz **(B)**, and 4 Hz **(C)** frequency separations between the two singlets, the correlation coefficient was found to be −0.04, −0.63, and −0.98, respectively.

Many neurometabolites have similar resonant frequencies, leading to spectral overlap among MRS signals. Effects of spectral overlap on the Cremer–Rao lower bounds of extracted neurometabolites have been analyzed previously (104). Extracting the concentrations of neurometabolites by spectral fitting is essentially mapping the acquired MRS spectrum (**spec**) into a vector (**conc**) consisting of concentrations by inverting the matrix equation **spec** = **basis** • **conc** (24). Here, **basis** is a matrix consisting of basis spectra of the component neurometabolites, which transforms the concentration vector **conc** into the fitted spectrum that approximates **spec** in the sense of least squares. Overlapping neurometabolites become statistically correlated through the covariance matrix (COV) of **conc** (105):

$$\text{COV}(\mathrm{conc})=\sigma^{2}{\left(\mathrm{basi}s^{†}•\;\mathrm{basis}\right)}^{-1}$$

where ^†^ denotes Hermitian transposition and *σ*^2^ is the noise variance of the measured spectrum **spec**. The off-diagonal elements (proportional to the square of cross-correlation coefficients) of COV(**conc**) depend on the frequency separation among the resonances of the neurometabolites in **basis**. **Figure 2** shows Monte Carlo simulations of the correlation between two singlet peaks as a function of their separation in the frequency domain. **Figure 2A** shows the correlation between conc_1_ and conc_2_ across individual fits when spectral overlap between the two signals is minimal. The correlation between conc_1_ and conc_2_ when their frequency separation equals their half-height linewidth is plotted in **Figure 2B**. Finally, the correlation between conc_1_ and conc_2_ when their frequency separation equals 0.5* half-height linewidth is plotted in **Figure 2C**. As shown by **Figure 2** statistical correlation between these two neurochemically unrelated signals increases as their spectral overlap increases. Here the large correlation values occur when the two signals happen to exhibit similar chemical shifts, not because one signal influences the other neurochemically. This simple example of two overlapping singlets illustrates a point of caution in the interpretation of neurometabolite correlation results. For multiplets and neurometabolites with multiple resonances, spectral overlap occurs when two resonance lines overlap each other even when the chemical shifts are not very close.

**Figure 3:**
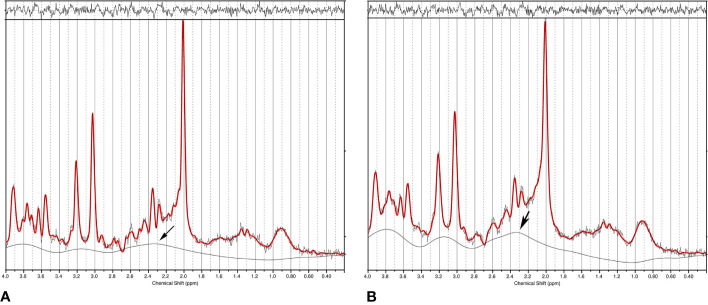
**(A)** Single voxel short echo time spectrum acquired from the anterior cingulate cortex of a healthy subject at 3 T. Data was fitted using LCModel. **(B)** LCModel generated a different baseline from the same data after 2.0 Hz line-broadening and noise injection to maintain the same signal-to-noise ratio. The large change in baseline around 2.35 ppm (marked by an arrow) simultaneously reduced fitted NAA and glutamate concentrations, therefore, causing positive correlation between NAA and glutamate even though the only difference between the two spectra is their linewidth (*i.e.*, no correlations). Reprinted from reference (107) with permission from Elsevier.

The off-diagonal elements of the covariance matrix become highly significant in the presence of severe spectral overlap such as in short echo time MRS spectra. In addition to overlapping neurometabolites, a strong baseline can also cause statistical correlations (106, 107) because the baseline, which arises from macromolecules and/or lipids and residual water, overlaps with essentially all neurometabolite signals. **Figure 3** compares spectral fitting of two 3 T short echo time single voxel *in vivo* spectra by the commercial LCModel software. The spectrum on the left was acquired using short echo time Point RESolved Spectroscopy (PRESS) technique from a cubic voxel in the anterior cingulate cortex of a healthy subject at 3 T (echo time = 35 ms, voxel size = 8 ml). The spectrum on the right was generated by broadening the linewidths of the spectrum on the left by 2.0 Hz and adding random noise to maintain the same signal-to-noise ratio (107). Both spectra in **Figure 3** were fitted using LCModel with the same default settings (24). With 2.0 Hz line-broadening the LCModel baseline was conspicuously stronger around the spectral region near 2.35 ppm where glutamate and the aspartyl moiety of NAA resonate. Both NAA and glutamate levels reported by LCModel were lowered by approximately the same amount (~11%) after the line-broadening [n = 10; (107)]. This reduction in the extracted metabolite concentrations was found to be generally more pronounced with greater line-broadening. Although the concentrations of neurometabolites in the two spectra of **Figure 3** are identical, the LCModel produced lower neurometabolite levels and a more intense baseline after 2.0 Hz line-broadening. Because of the spectral overlap between baseline and neurometabolites, overestimating (underestimating) the baseline causes underestimating (overestimating) neurometabolites and *vice versa*. Neurometabolites overlapping with the same broad baseline peak are similarly underestimated (overestimated) due to the broad baseline signals. This in turn, contributes to positive statistical correlations among those neurometabolites regardless of the underlying neurochemical associations.

Spectral fitting techniques such as the LCModel heavily rely on the linewidth difference between neurometabolites and background signals to separate them. Broad neurometabolite peaks in the presence of a strong baseline, as often seen in clinical short echo time MRS data, can lead to significant quantification errors and unwanted statistical correlations because of the large baseline-metabolite covariances. The results in **Figure 3** are also corroborated by an earlier study which quantitatively analyzed the estimation uncertainties caused by the baseline using Cramer–Rao lower bound (CRLB) of the baseline (106), confirming that the estimation uncertainty significantly increases with decreased baseline smoothness and increased spectral linewidths.

## Prospects for Mapping Macroscopic Neurochemical Associations By MRS Imaging

Although group comparison of neurometabolite correlations between healthy controls and patients reveals altered neurochemical associations in brain disorders, determining the absolute strength of these correlations is important for interpreting clinical findings (20, 21) and for potentially relaying interregional associations across the brain. As linewidth variations in clinical MRS studies are very common, our analysis of statistical correlations that originated from the spectral overlap in the section *Statistical Correlations Among MRS Signals Due to Spectral Overlap* has demonstrated that spectral overlap should be eliminated or minimized when the absolute strength of neurometabolite correlations is to be determined. To facilitate measurement of the absolute strength of neurochemical associations, MRS techniques that result in flat baselines and isolated signals of interest would be ideal. Many existing single voxel spectral editing techniques generate flat or weak baselines while eliminating or minimizing overlapping resonances (74, 108–115), which are likely suited for measuring local or intraregional neurochemical associations.

Mapping macroscopic neurochemical associations across the human brain has the exciting potential to broadly impact studies of normal brain functions as well as neuropsychiatric disorders (32, 116, 117). Here we discuss the prospects for measuring interregional excitation–inhibition balance (32, 118–120) by spectroscopic imaging of spectrally resolved glutamate and GABA. Participant motion is a major issue in scanning many patients of neuropsychiatric disorders. Studying these patients using chemical shift imaging is technically challenging because of the relatively long scan time required for phase encoding. As artifacts in chemical shift images caused by motion are hard to detect, they can lead to erroneous diagnosis and data interpretation (121). It is well-known that participant motion inside a magnetic field causes changes in resonant frequencies. Incorporating spectral editing techniques based on highly selective radiofrequency pulses into chemical shift imaging therefore can lead to even larger errors due to the additional effects of carrier frequency mismatch on spectral editing yield.

To minimize error due to unavoidable patient movement during extended scan time necessary for phase encoding, we focus on techniques that can resolve glutamate or GABA in a single shot with relatively weak baselines at 7 T. Spectral isolation of glutamate or GABA accompanied with a weak baseline will minimize the unwanted correlations that originated from spectral overlap (107). The emphasis on minimizing spectral overlap for measuring interregional neurometabolite correlations may seem counterintuitive. However, it is necessary because, for example, overlapping with interregionally correlated signals can relay the correlation to overlapped signals. To spectrally resolve glutamate or GABA over an extended brain region, highly frequency-selective pulses popular for single voxel spectral editing cannot be used because of the unavoidable and significant residual B_0_ inhomogeneity across a large volume in the brain, especially at high magnetic field strength. Highly frequency-selective pulses are sensitive to patient movement, system instability, and B_0_ inhomogeneity as they will miss or partially miss the editing target in part of the slice(s) where resonance frequencies are shifted away (122, 123).

**Figure 4:**
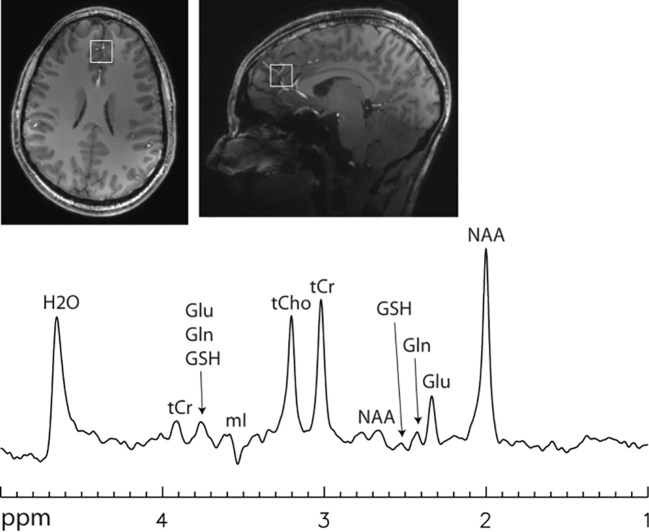
Single voxel 7 T spectrum acquired using a single-shot echo time optimized PRESS sequence (112) without signal averaging. Voxel size = 2 × 2 × 2 cm^3^. Echo time = 106 ms. Line broadening = 8 Hz. Number of averages = 1. NAA, N-acetylaspartate; Glu, glutamate; Gln, glutamine; GSH, glutathione; tCr, total creatine; tCho, total choline; mI, myo-inositol. Glutamate H4 at 2.35 ppm was spectrally resolved with an approximately flat baseline.

Weak or nearly flat baselines are automatically produced at long echo times because of the shorter T_2_ values of macromolecules (1). Serendipitously, the strongly coupled glutamate H4 (2.35 ppm) forms an intense pseudo singlet at a relatively long echo time (~100 ms) at 7 T (111, 112). The resonances of glutamine H4 at 2.45 ppm and glutathione glutamyl H4 at 2.49 ppm also form pseudo singlets at ~100 ms echo time (see **Figure 4**). Therefore, glutamate can be spectrally resolved in a single shot without using any spectrally selective pulses at ~100 ms echo time at 7 T (111). At 7 T the multiplet signal of the aspartyl moiety of NAA at 2.49 ppm still overlaps with glutamine H4 and glutathione glutamyl H4 at ~100 ms echo time (111). The overlapping NAA aspartyl moiety signals can be eliminated using a J-suppression pulse acting on the *α*-H of the aspartyl moiety of NAA at 4.38 ppm (112). The J suppression pulse can be made band-selective with a flat top frequency profile (124) to accommodate variations in B_0_. Either because no frequency selective pulses are needed (111) or with band-selective J suppression (112), chemical shift imaging of glutamate is feasible at 7 T in the presence of significant patient motion and residual static magnetic field inhomogeneity across the slice(s).

**Figure 5:**
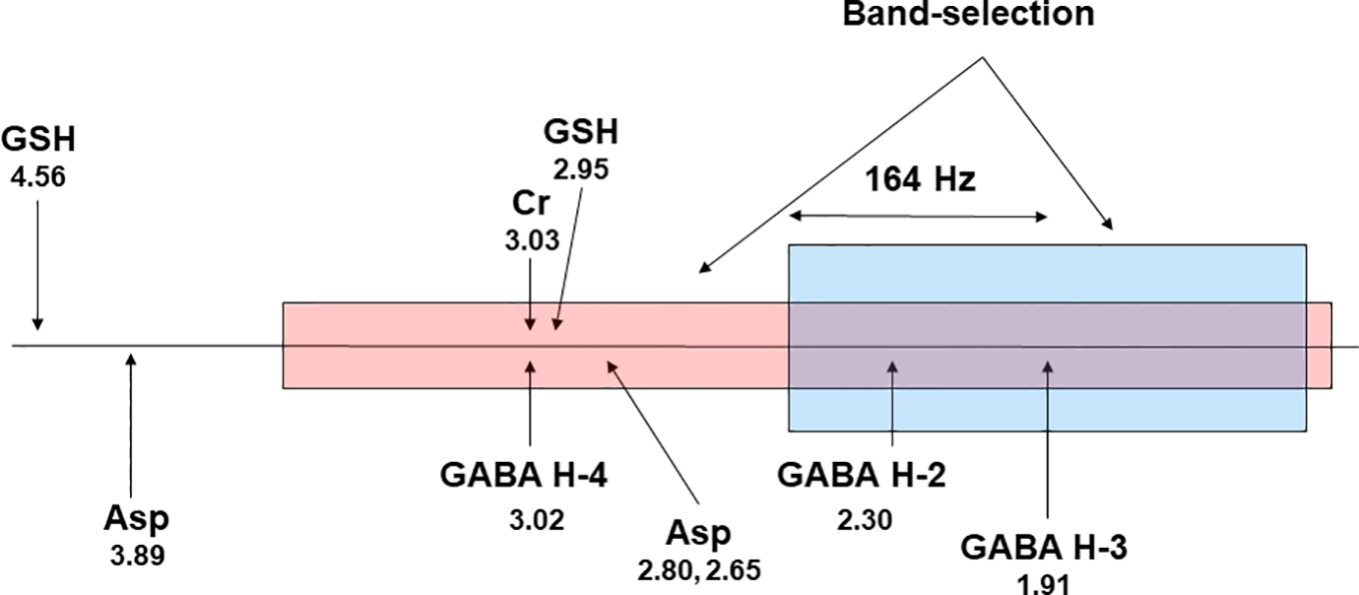
A proposed scheme for band-selective multiple quantum filtering of GABA. A band-selective refocusing pulse prepares GABA into multiple quantum state while leaving glutathione in single quantum state by avoiding refocusing its cysteinyl *α*-H at 4.56 ppm. A band-selective 90^°^ pulse converts the double quantum coherence into observable single quantum coherence. The frequency separation between GABA H4 and GABA H3 at 7 T is 164 × 2 = 328 Hz. Asp, aspartate; GSH, glutathione; Cr, creatine. The chemical shifts (in ppm) of the resonance signals are placed below their labels.

Multiple quantum filtering can be used to edit GABA in a single shot (125–128). It is also possible to generate a flat baseline using multiple quantum filtering (127). Chemical shift imaging of GABA over a large volume in the brain is challenging even at the high magnetic field strength of 7 T as all available GABA editing techniques rely on frequency selective pulses that differentially act on GABA H3 at 1.91 ppm and GABA H4 at 3.02 ppm. At 7 T, the chemical shift dispersion between GABA H3 and GABA H4 is 328 Hz. This relatively large chemical shift difference makes it possible to use band-selective pulses with a flat top (124) to accommodate changes in B_0_ due to patient movement, system instability, and residual B_0_ inhomogeneity while still affording a close to uniform editing yield. **Figure 5** proposes a band-selective multiple quantum filtering scheme that combines spectral selectivity while allowing signal to shift within the flat passband. This multiple quantum GABA editing scheme is expected to tolerate a large frequency shift with GABA H3 lying within the flat bandwidth and GABA H4 staying outside of the downfield transition band of the band-selective pulse acting on GABA H3.

Although we have focused on discussing chemical shift imaging of spectrally resolved glutamate and GABA without using highly selective editing pulses for the magnetic field strength of 7 T, similar ideas may also be developed for lower magnetic field strengths such as 3 T. For example, multiecho time averaging at 3 T can isolate glutamate with a nearly flat baseline without using any spectral editing pulses (109). Directly combining this approach with conventional phase-encoding for chemical shift imaging would not be feasible due to the large number [e.g., (32)] of echoes required for each phase encoding step. Instead of using evenly spaced echo times, the averaging effect may be obtained using fewer echo times with numerical optimization. Many fast imaging strategies such as echo-planar readout can also greatly accelerate data acquisition of chemical shift imaging experiments.

The coordinated variations of glutamate and GABA across the brain can be assessed using chemical shift images of spectrally resolved glutamate and GABA. The absolute strengths of interregional correlations of spectrally resolved glutamate and GABA have the exciting potential for characterizing excitatory–inhibitory connections among the nodes of neural networks, therefore providing novel parameters for gauging interregional excitation–inhibition balance, the disruption of which is implicated in many neuropsychiatric disorders [e.g., (33, 103, 117, 129, 130)].

## Conclusions

Previous neurochemical studies of animal models have revealed coordinated changes in major neurometabolites under various pathophysiological conditions which were attributed to the well-studied metabolic pathways connecting them. Recent MRS findings of neurometabolite correlations in healthy subjects and altered correlations in patients have further corroborated these associations, and their disruption is a hallmark of many neuropsychiatric disorders. To measure the absolute strength of these correlations, it is necessary to use spectral editing techniques to minimize or eliminate statistical correlations among MRS signals that originated from spectral overlap. Finally, chemical shift imaging of spectrally resolved glutamate and GABA is technically feasible at 7 T. It is hoped that the prospects for eliminating the confounding statistical correlations due to spectral overlap will reduce controversies in the field and generate further interest in characterizing local and interregional neurochemical associations especially glutamate–GABA interactions in the brain for studying neuropsychiatric disorders.

## Author Contributions

JS conceived the paper and performed literature search. DS and LA conducted laboratory research. JST performed literature search. JS, LA, and JST wrote the paper. All authors contributed to the article and approved the submitted version.

## Funding

This work was supported by the Intramural Research Program of National Institute of Mental Health, NIH.

## Conflict of Interest

The authors declare that the research was conducted in the absence of any commercial or financial relationships that could be construed as a potential conflict of interest.

## References

[1] de Graaf RA In vivo NMR spectroscopy. John Wiley & Sons: Chichester, UK (1998).

[2] Kleinhans NM Schweinsburg BC Cohen DN Müller RA Courchesne E N-acetyl aspartate in autism spectrum disorders: regional effects and relationship to fMRI activation. Brain Res (2007) 1162:85–97. 10.1016/j.brainres.2007.04.081 17612510 PMC3477551

[3] Walter M Henning A Grimm S Schulte RF Beck J Dydak U The relationship between aberrant neuronal activation in the pregenual anterior cingulate, altered glutamatergic metabolism, and anhedonia in major depression. Arch Gen Psychiatry (2009) 66:478–86. 10.1001/archgenpsychiatry.2009.39 19414707

[4] Sudharshan N Hanstock C Hui B Pyra T Johnston W Kalra S Degeneration of the mid-cingulate cortex in amyotrophic lateral sclerosis detected in vivo with MR spectroscopy. AJNR Am J Neuroradiol (2011) 32:403–7. 10.3174/ajnr.A2289 PMC7965735 21087934

[5] Waddell KW Zanjanipour P Pradhan S Xu L Welch EB Joers JM Anterior cingulate and cerebellar GABA and Glu correlations measured by ¹H J-difference spectroscopy. Magn Reson Imaging (2011) 29:19–24. 10.1016/j.mri.2010.07.005 20884148 PMC3005886

[6] Kraguljac NV Reid MA White DM den Hollander J Lahti AC Regional decoupling of N-acetyl-aspartate and glutamate in schizophrenia. Neuropsychopharmacology (2012) 37:2635–42. 10.1038/npp.2012.126 PMC3473328 22805603

[7] Kraguljac NV White DM Reid MA Lahti AC Increased hippocampal glutamate and volumetric deficits in unmedicated patients with schizophrenia. JAMA Psychiatry (2013) 70:1294–302. 10.1001/jamapsychiatry.2013.2437 PMC7891898 24108440

[8] Gussew A Borys C Janetzki L Cleve M Malessa R Habenicht U Altered regional and interregional interrelations of glutamate and GABA in patients with chronic low back pain – A **^1^**H-MR spectroscopic study, Clin. Neurophysiology (2015) 126:e109–10. 10.1016/j.clinph.2015.04.165

[9] Plitman E de la Fuente-Sandoval C Reyes-Madrigal F Chavez S Gómez-Cruz G León-Ortiz P Elevated myo-inositol, choline, and glutamate levels in the associative striatum of antipsychotic-naive patients with first-episode psychosis: a proton magnetic resonance spectroscopy study with implications for glial dysfunction. Schizophr Bull (2016) 42:415–24. 10.1093/schbul/sbv118 PMC4753594 26320195

[10] Cleve M Gussew A Wagner G Bär KJ Reichenbach JR Assessment of intra- and inter-regional interrelations between GABA+, Glx and BOLD during pain perception in the human brain - A combined ^1^H fMRS and fMRI study. Neuroscience (2017) 365:125–36. 10.1016/j.neuroscience.2017.09.037 28965838

[11] Sala A Caminiti SP Presotto L Premi E Pilotto A Turrone R Altered brain metabolic connectivity at multiscale level in early Parkinson's disease. Sci Rep (2017) 7:4256. 10.1038/s41598-017-04102-z 28652595 PMC5484707

[12] Niddam DM Lai KL Tsai SY Lin YR Chen WT Fuh JL Neurochemical changes in the medial wall of the brain in chronic migraine. Brain (2018) 141:377–90. 10.1093/brain/awx331 29236991

[13] Taylor-Robinson SD Buckley C Changani KK Hodgson HJ Bell JD Cerebral proton and phosphorus-31 magnetic resonance spectroscopy in patients with subclinical hepatic encephalopathy. Liver (1999) 19:389–98. 10.1111/j.1478-3231.1999.tb00067.x 10533796

[14] Grachev ID Apkarian AV Chemical network of the living human brain. Evidence of reorganization with aging. Brain Res Cognit Brain Res (2001) 11:185–97. 10.1016/S0926-6410(00)00068-9 11275481

[15] Cleve M Gussew A Janetzki L Borys C Reichenbach JR Interregional associations between excitatory and inhibitory neurotransmitters in the resting human brain. Proc ISMRM (2015) 2348.

[16] Just N Sonnay S Investigating the Role of Glutamate and GABA in the Modulation of Transthalamic Activity: A Combined fMRI-fMRS Study. Front Physiol (2017) 8:30. 10.3389/fphys.2017.00030 28197105 PMC5281558

[17] van Veenendaal TM Backes WH Tse DHY Scheenen TWJ Klomp DW Hofman PAM High field imaging of large-scale neurotransmitter networks: Proof of concept and initial application to epilepsy. NeuroImage Clin (2018) 19:47–55. 10.1016/j.nicl.2018.04.006 30035001 PMC6051471

[18] Kim HJ Kim JE Cho G IC S Bae S SJ H Associations between anterior cingulate cortex glutamate and gamma-aminobutyric acid concentrations and the harm avoidance temperament. Neurosci Lett (2009) 464:103–7. 10.1016/j.neulet.2009.07.087 19660524

[19] Tremblay S Beaulé V Proulx S de Beaumont L Marjanska M Doyon J Relationship between transcranial magnetic stimulation measures of intracortical inhibition and spectroscopy measures of GABA and glutamate+glutamine. J Neurophysiol (2013) 109:1343–9. 10.1152/jn.00704.2012 PMC3602833 23221412

[20] Kraguljac NV Cutter GR Morgan C Lahti AC In reply. JAMA Psychiatry (2014) 71:339–40. 10.1001/jamapsychiatry.2014.22 24599245

[21] Maddock R The problem of spurious correlations between pairs of brain metabolite values measured in the same voxel with magnetic resonance spectroscopy. JAMA Psychiatry (2014) 71:338–9. 10.1001/jamapsychiatry.2013.4343 24599243

[22] Ende G Cackowski S Van Eijk J Sack M Demirakca T Kleindienst N Impulsivity and aggression in female BPD and ADHD patients: association with ACC glutamate and GABA concentrations. Neuropsycho-pharmacology (2016) 41:410–8. 10.1038/npp.2015.153 PMC5130117 26040503

[23] Scholl J Kolling N Nelissen N Stagg CJ Harmer CJ Rushworth MF Excitation and inhibition in anterior cingulate predict use of past experiences. elife (2017) 6:e20365. 10.7554/eLife.20365 28055824 PMC5213710

[24] Provencher SW Estimation of Metabolite Concentrations from Localized in-Vivo Proton Nmr Spectra. Magn Reson Med (1993) 30(6):672–9. 10.1002/mrm.1910300604 8139448

[25] Siesjö BK Brain Energy Metabolism. John Wiley & Sons: Chichester, UK (1978).

[26] Erecinska M Silver IA Metabolism and role of glutamate in mammalian brain. Prog Neurobiol (1990) 35:245–96. 10.1016/0301-0082(90)90013-7 1980745

[27] Watts ME Pocock R Claudianos C Brain energy and oxygen metabolism: emerging role in normal function and disease. Front Mol Neurosci (2018) 11:216. 10.3389/fnmol.2018.00216 29988368 PMC6023993

[28] Duncan NW Wiebking C Northoff G Associations of regional GABA and glutamate with intrinsic and extrinsic neural activity in humans - a review of multimodal imaging studies. Neurosci Biobehav Rev (2014) 47:36–52. 10.1016/j.neubiorev.2014.07.016 25066091

[29] Ajram LA Pereira AC Durieux AMS Velthius HE Petrinovic MM McAlonan GM The contribution of [^1^H] magnetic resonance spectroscopy to the study of excitation-inhibition in autism. Prog Neuropsychopharmacol Biol Psychiatry (2019) 89:236–44. 10.1016/j.pnpbp.2018.09.010 30248378

[30] Yildiz-Yesiloglu A Ankerst DP Review of ^1^H magnetic resonance spectroscopy findings in major depressive disorder: a meta-analysis. Psychiatry Res (2006) 147:1–25. 10.1016/j.pscychresns.2005.12.004 16806850

[31] Duman RS Sanacora G Krystal JH Altered connectivity in depression: GABA and glutamate neurotransmitter deficits and reversal by novel treatments. Neuron (2019) 102:75–90. 10.1016/j.neuron.2019.03.013 30946828 PMC6450409

[32] Sohal VS Rubenstein JLR Excitation-inhibition balance as a framework for investigating mechanisms in neuropsychiatric disorders. Mol Psychiatry (2019) 24:1248–57. 10.1038/s41380-019-0426-0 PMC6742424 31089192

[33] Hjelmervik H Craven AR Sinceviciute I Johnsen E Kompus K Bless JJ Intra-Regional Glu-GABA vs Inter-Regional Glu-Glu Imbalance: A ^1^H-MRS Study of the Neurochemistry of Auditory Verbal Hallucinations in Schizophrenia. Schizophr Bull (2020) 46:633–42. 10.1093/schbul/sbz099 PMC7147588 31626702

[34] Bruhn H Frahm J Gyngell ML Merboldt KD Hanicke W Sauter R Cerebral metabolism in man after acute stroke: new observations using localized proton NMR spectroscopy. Magn Reson Med (1989) 9:126–31. 10.1002/mrm.1910090115 2540394

[35] Lemesle M Walker P Guy F D'Athis P Billiar T Giroud M Multi-variate analysis predicts clinical outcome 30 days after middle cerebral artery infarction. Acta Neurol Scand (2000) 102:1–17. 10.1034/j.1600-0404.2000.102001011.x 10893057

[36] Moffett JR Ross B Arun P Madhavarao CN Namboodiri AM N-Acetylaspartate in the CNS: from neurodiagnostics to neurobiology. Prog Neurobiol (2007) 81:89–131. 10.1016/j.pneurobio.2006.12.003 17275978 PMC1919520

[37] Magistretti PJ Cellular bases of functional brain imaging: insights from neuron-glia metabolic coupling. Brain Res (2000) 886:108–12. 10.1016/S0006-8993(00)02945-0 11119692

[38] Rothman DL de Graaf RA Hyder F Mason GF Behar KL De Feyter HM In vivo ^13^C and ^1^H-[^13^C] MRS studies of neuroenergetics and neurotransmitter cycling, applications to neurological and psychiatric disease and brain cancer. NMR Biomed (2019) 32:e4172. 10.1002/nbm.4172 31478594

[39] Srinivasan R Sailasuta N Hurd R Nelson S Daniel Pelletier D Evidence of elevated glutamate in multiple sclerosis using magnetic resonance spectroscopy at 3 T. Brain (2005) 128:1016–25. 10.1093/brain/awh467 15758036

[40] Hasler G van der Veen JW Tumonis T Meyers N Shen J Drevets WC Reduced prefrontal glutamate/glutamine and gamma-aminobutyric acid levels in major depression determined using proton magnetic resonance spectroscopy. Arch Gen Psychiatry (2007) 64:193–200. 10.1001/archpsyc.64.2.193 17283286

[41] Strawn JR Patel NC Chu W-J Lee J-H Adler CM Kim M-J Glutamatergic effects of divalproex in adolescents with mania: a proton magnetic resonance spectroscopy study. J Am Acad Child Adolesc Psychiatry (2012) 51:642–51. 10.1016/j.jaac.2012.03.009 PMC4499458 22632623

[42] Yelamanchi SD Jayaram S Thomas JK Gundimeda S Khan AA Singhal A A pathway map of glutamate metabolism. J Cell Commun Signal (2016) 10:69–75. 10.1007/s12079-015-0315-5 26635200 PMC4850134

[43] Shen J In vivo carbon-13 magnetization transfer effect. Detection of aspartate aminotransferase reaction. Magn Reson Med (2005) 54:1321–6. 10.1002/mrm.20709 16270328

[44] Lewis LD Ljunggren B Norberg K Siesjö BK Changes in carbohydrate substrates, amino acids and ammonia in the brain during insulin-induced hypoglycemia. J Neurochem (1974) 23:659–71. 10.1111/j.1471-4159.1974.tb04389.x 4154353

[45] Engelsen B Fonnum F Effects of hypoglycemia on the transmitter pool and the metabolic pool of glutamate in rat brain. Neurosci Lett (1983) 42:317–22. 10.1016/0304-3940(83)90281-1 6141542

[46] Wong KL Tyce GM Glucose and amino acid metabolism in rat brain during sustained hypoglycemia. Neurochem Res (1983) 8:401–15. 10.1007/BF00965097 6888644

[47] Betz AL Gilboe DD Effect of pentobarbital on amino acid and urea flux in the isolated dog brain. Am J Physiol (1973) 224:580–7. 10.1152/ajplegacy.1973.224.3.580 4691272

[48] Hägerdal M Harp J Siesjö BK Effect of hypothermia upon organic phosphates, glycolytic metabolites, citric acid cycle intermediates and associated amino acids in rat cerebral cortex. J Neurochem (1975) 24:743–8. 10.1111/j.1471-4159.1975.tb11673.x 1123628

[49] Chapman AG Nordström CH Siesjö BK Influence of phenobarbital anesthesia on carbohydrate and amino acid metabolism in rat brain. Anesthesiology (1978) 48:175–82. 10.1097/00000542-197803000-00003 626422

[50] MacMillan V Siesjö BK The influence of hypocapnia upon intracellular pH and upon some carbohydrate substrates, amino acids and organic phosphates in the brain. J Neurochem (1973) 21:1283–99. 10.1111/j.1471-4159.1973.tb07582.x 4761709

[51] Patel TB Clark JB Synthesis of N-acetyl-L-aspartate by rat brain mitochondria and its involvement in mitochondrial/cytosolic carbon transport. Biochem J (1979) 184(3):539–46. 10.1042/bj1840539 PMC1161835 540047

[52] Robinson MB Blakely RD Couto R Coyle JT Hydrolysis of the brain dipeptide N-acetyl-L-aspartyl-L-glutamate. Identification and characterization of a novel N-acetylated alpha-linked acidic dipeptidase activity from rat brain. J Biol Chem (1987) 262:14498–506. 3667587

[53] Bzdega T Turi T Wroblewska B She D Chung HS Kim H Molecular cloning of a peptidase against N-acetylaspartylglutamate from a rat hippocampal cDNA library. J Neurochem (1997) 69(6):2270–7. 10.1046/j.1471-4159.1997.69062270.x 9375657

[54] Luthi-Carter R Berger UV Barczak AK Enna M Coyle JT Isolation and expression of a rat brain cDNA encoding glutamate carboxypeptidase II. Proc Natl Acad Sci USA (1998) 95:3215–20. 10.1073/pnas.95.6.3215 PMC19722 9501243

[55] Clark JF Doepke A Filosa JA Wardle RL Lu A Meeker TJ N-acetylaspartate as a reservoir for glutamate. Med Hypotheses (2006) 67:506–12. 10.1016/j.mehy.2006.02.047 16730130

[56] Chavarria L Cordoba J Magnetic resonance imaging and spectroscopy in hepatic encephalopathy. J Clin Exp Hepatol (2015) 5:S69–74. 10.1016/j.jceh.2013.10.001 PMC4442861 26041961

[57] Natarajan SK Venneti S Glutamine metabolism in brain tumors. Cancers (2019) 11:1628. 10.3390/cancers11111628 PMC6893651 31652923

[58] Sibson NR Dhankhar A Mason GF Behar KL Rothman DL Shulman RG In vivo ^13^C NMR measurements of cerebral glutamine synthesis as evidence for glutamate-glutamine cycling. Proc Natl Acad Sci U S A (1997) 94:2699–704. 10.1073/pnas.94.6.2699 PMC20152 9122259

[59] Rothman DL De Feyter HM de Graaf RA Mason GF Behar KL ^13^C MRS studies of neuroenergetics and neurotransmitter cycling in humans. NMR Biomed (2011) 24:943–57. 10.1002/nbm.1772 PMC3651027 21882281

[60] Greene JG Greenamyre JT Bioenergetics and glutamate excitotoxicity. Prog Neurobiol (1996) 48:613–34. 10.1016/0301-0082(96)00006-8 8809910

[61] Rothstein JD Dykes-Hoberg M Pardo CA Bristol LA Jin L Kuncl RW Knockout of glutamate transporters reveals a major role for astroglial transport in excitotoxicity and clearance of glutamate. Neuron (1996) 16:675–86. 10.1016/S0896-6273(00)80086-0 8785064

[62] Gether U Andersen PH Larsson OM Schousboe A Neurotransmitter transporters: molecular function of important drug targets. Trends Pharmacol Sci (2006) 27:375–83. 10.1016/j.tips.2006.05.003 16762425

[63] Hertz L Intercellular metabolic compartmentation in the brain. Past Present Future Neurochem Int (2004) 45:285–96. 10.1016/j.neuint.2003.08.016 15145544

[64] Duce IR Keen P Selective uptake of [^3^H]glutamine and [^3^H]glutamate into neurons and satellite cells of dorsal root ganglia in vitro. Neuroscience (1983) 8:861–6. 10.1016/0306-4522(83)90016-7 6866267

[65] Sibson NR Mason GF Shen J Cline GW Herskovits AZ Wall JE In vivo ^13^C NMR measurement of neurotransmitter glutamate cycling, anaplerosis and TCA cycle flux in rat brain during. J Neurochem (2001) 76:975–89. 10.1046/j.1471-4159.2001.00074.x 11181817

[66] Gruetter R Seaquist ER Kim S Ugurbil K Localized in vivo ^13^C-NMR of glutamate metabolism in the human brain: initial results at 4 Tesla. Dev Neurosci (1998) 20:380–8. 10.1159/000017334 9778575

[67] Shen J Sibson NR Cline G Behar KL Rothman DL Shulman RG ^15^N-NMR spectroscopy studies of ammonia transport and glutamine synthesis in the hyperammonemic rat brain. Dev Neurosci (1998) 20:434–43. 10.1159/000017341 9778582

[68] Shen J Petersen KF Behar KL Brown P Nixon TW Mason GF Determination of the rate of the glutamate/glutamine cycle in the human brain by in vivo ^13^C NMR. Proc Natl Acad Sci USA (1999) 96:8235–40. 10.1073/pnas.96.14.8235 PMC22218 10393978

[69] Lebon V Petersen KF Cline GW Shen J Mason GF Dufour S Astroglial contribution to brain energy metabolism in humans revealed by ^13^C nuclear magnetic resonance spectroscopy. Elucidation of the dominant pathway for neurotransmitter glutamate repletion and measurement of astrocytic oxidative metabolism. J Neurosci (2002) 22:1523–31. 10.1523/JNEUROSCI.22-05-01523.2002 PMC2995528 11880482

[70] Shen J Modeling the glutamate-glutamine neurotransmitter cycle. Front Neuroenergetics (2013) 5(1). 10.3389/fnene.2013 PMC3556573 23372548

[71] Bradford HF Ward HK Thomas AJ Glutamine — a major substrate for nerve endings. J Neurochem (1978) 30:1453–9. 10.1111/j.1471-4159.1978.tb10477.x 670985

[72] de Graaf AA Deutz NE Bosman DK Chamuleau RA de Haan JG Bovee WM The use of in vivo proton NMR to study the effects of hyperammonemia in the rat cerebral cortex. NMR Biomed (1991) 4:31–7. 10.1002/nbm.1940040106 1674207

[73] Stelmashook EV Isaev NK Lozier ER Goryacheva ES Khaspekov LG Role of glutamine in neuronal survival and death during brain ischemia and hypoglycemia. Int J Neurosci (2011) 121:415–22. 10.3109/00207454.2011.570464 21574892

[74] Rothman DL Petroff OA Behar KL Mattson RH Localized ^1^H NMR measurements of gamma-aminobutyric acid in human brain in vivo. Proc Nat Acad Sci USA (1993) 90:5662–6. 10.1073/pnas.90.12.5662 PMC46781 8516315

[75] Petroff OA Behar KL Mattson RH Rothman DL Human brain gamma-aminobutyric acid levels and seizure control following initiation of vigabatrin therapy. J Neurochem (1996) 67:2399–404. 10.1046/j.1471-4159.1996.67062399.x 8931472

[76] Roberts E Frankel S γ-Aminobutyric acid in brain: Its formation from glutamic acid. J Biol Chem (1950) 187:55–63. 14794689

[77] Price NC Stevens L Fundamentals of Enzymology, 3rd ed. Oxford University Press: Oxford, UK (1999).

[78] Preece NE Cerdán S Metabolic precursors and compartmentation of cerebral GABA in vigabatrin-treated rats. J Neurochem (1996) 67:1718–25. 10.1046/j.1471-4159.1996.67041718.x 8858958

[79] Olsen RW DeLorey TM GABA Synthesis, Uptake and Release. In: Siegel GJ Agranoff BW Albers RW Fisher SK Uhler MD, editors. Basic Neurochemistry: Molecular, Cellular and Medical Aspects, 6th ed. Philadelphia, USA: Lippincott-Raven (1999).

[80] Yang J Li SS Bacher J Shen J Quantification of cortical GABA-glutamine cycling rate using in vivo magnetic resonance signal of [2-^13^C]GABA derived from glia-specific substrate [2-^13^C]acetate. Neurochem Int (2007) 50:371–8. 10.1016/j.neuint.2006.09.011 17056156

[81] Sequerra EB Gardino P Hedin-Pereira C de Mello FG Putrescine as an important source of GABA in the postnatal rat subventricular zone. Neuroscience (2007) 146:489–93. 10.1016/j.neuroscience.2007.01.062 17395389

[82] Tatti R Haley MS Swanson OK Tselha T Maffei A Neurophysiology and regulation of the balance between excitation and inhibition in neocortical circuits. Biol Psychiatry (2017) 81:821–31. 10.1016/j.biopsych.2016.09.017 PMC5374043 27865453

[83] Shepherd GM Neurobiology. 3rd ed. Oxford University Press: Oxford, UK (1994).

[84] Rubenstein JL Merzenich MM Model of autism: increased ratio of excitation/inhibition in key neural systems. Genes Brain Behav (2003) 2:255–67. 10.1034/j.1601-183X.2003.00037.x PMC6748642 14606691

[85] Kurcyus K Annac E Hanning NM Harris AD Oeltzschner G Edden R Opposite dynamics of GABA and glutamate levels in the occipital cortex during visual processing. J Neurosci (2018) 38:9967–76. 10.1523/JNEUROSCI.1214-18.2018 PMC6234295 30282724

[86] Maddock RJ Casazza GA Fernandez DH Maddock MI Acute modulation of cortical glutamate and GABA content by physical activity. J Neurosci (2016) 36:2449–57. 10.1523/JNEUROSCI.3455-15.2016 PMC6705493 26911692

[87] Harada M Taki MM Nose A Kubo H Mori K Nishitani H Non-invasive evaluation of the GABAergic/glutamatergic system in autistic patients observed by MEGA-editing proton MR spectroscopy using a clinical 3 tesla instrument. J Autism Dev Disord (2011) 41:447–54. 10.1007/s10803-010-1065-0 20652388

[88] Sanacora G Gueorguieva R Epperson CN Wu YT Appel M Rothman DL Subtype-specific alterations of gamma-aminobutyric acid and glutamate in patients with major depression. Arch Gen Psychiatry (2004) 61:705–13. 10.1001/archpsyc.61.7.705 15237082

[89] Pollack MH Jensen JE Simon NM Kaufman RE Renshaw PF High-field MRS study of GABA, glutamate and glutamine in social anxiety disorder: response to treatment with levetiracetam. Prog Neuropsychopharmacol Biol Psychiatry (2008) 32:739–43. 10.1016/j.pnpbp.2007.11.023 18206286

[90] Maddock RJ Buonocore MH MR spectroscopic studies of the brain in psychiatric disorders. Curr Top Behav Neurosci (2012) 11:199–251. 10.1007/7854_2011_197 22294088

[91] Thomson AM Lamy C Functional maps of neocortical local circuitry. Front Neurosci (2007) 1:19–42. 10.3389/neuro.01.1.1.002.2007 18982117 PMC2518047

[92] Caputi A Melzer S Michael M Monyer H The long and short of GABAergic neurons. Curr Opin Neurobiol (2013) 23:179–86. 10.1016/j.conb.2013.01.021 23394773

[93] Zikopoulos B Barbas H Altered neural connectivity in excitatory and inhibitory cortical circuits in autism. Front Hum Neurosci (2013) 7:609. 10.3389/fnhum.2013.00609 24098278 PMC3784686

[94] Wood JD Kurylo E Lane R gamma-Aminobutyric acid release from synaptosomes prepared from rats treated with isonicotinic acid hydrazide and gabaculine. J Neurochem (1988) 50:1839–43. 10.1111/j.1471-4159.1988.tb02486.x 3373216

[95] Golan H Talpalar AE Schleifstein-Attias D Grossman Y GABA metabolism controls inhibition efficacy in the mammalian CNS. Neurosci Lett (1996) 217:25–8. 10.1016/0304-3940(96)13061-5 8905731

[96] Jackson MF Esplin B Capek R Reversal of the activity-dependent suppression of GABA-mediated inhibition in hippocampal slices from gamma-vinyl GABA (vigabatrin)-pretreated rats. Neuropharmacology (2000) 39:65–74. 10.1016/S0028-3908(99)00075-1 10665820

[97] Wu Y Wang W Richerson GB GABA transaminase inhibition induces spontaneous and enhances depolarization-evoked GABA efflux via reversal of the GABA transporter. J Neurosci (2001) 21:2630–9. 10.1523/JNEUROSCI.21-08-02630.2001 PMC6762542 11306616

[98] Chen Z Silva AC Yang J Shen J Elevated endogenous GABA level correlates with decreased fMRI signals in the rat brain during acute inhibition of GABA transaminase. J Neurosci Res (2005) 79:383–91. 10.1002/jnr.20364 15619231

[99] Yang J Shen J Elevated endogenous GABA concentration attenuates glutamate-glutamine cycling between neurons and astroglia. J Neural Transm (2009) 116:291–300. 10.1007/s00702-009-0186-0 19184333 PMC2845912

[100] Rae CD A guide to the metabolic pathways and function of metabolites observed in human brain ^1^H magnetic resonance spectra. Neurochem Res (2014) 39:1–36. 10.1007/s11064-013-1199-5 24258018

[101] Boumezbeur F Mason GF de Graaf RA Behar KL Cline GW Shulman GI Altered brain mitochondrial metabolism in healthy aging as assessed by in vivo magnetic resonance spectroscopy. J Cereb Blood Flow Metab (2010) 30:211–21. 10.1038/jcbfm.2009.197 PMC2949111 19794401

[102] Wagner G Gussew A Köhler S de la Cruz F Smesny S Reichenbach JR Resting state functional connectivity of the hippocampus along the anterior-posterior axis and its association with glutamatergic metabolism. Cortex (2016) 81:104–17. 10.1016/j.cortex.2016.03.022 27182810

[103] Shukla DK Wijtenburg SA Chen H Chiappelli JJ Kochunov P Hong LE Anterior cingulate glutamate and GABA associations on functional connectivity in schizophrenia. Schizophr Bull (2019) 45:647–58. 10.1093/schbul/sby075 PMC6483591 29912445

[104] Cavassila S Deval S Huegen C van Ormondt D Graveron-Demilly D Cramer-Rao bounds an evaluation tool for quantitation. NMR Biomed (2001) 14:278–83. 10.1002/nbm.701 11410946

[105] Draper NR Smith H Applied regression analysis. 3rd ed. Wiley, New York: USA (1998). p. 135–139.

[106] Zhang Y Shen J Smoothness of in vivo spectral baseline determined by mean-square error. Magn Reson Med (2014) 72:913–22. 10.1002/mrm.25013 PMC4028435 24259436

[107] Zhang Y Shen J Effects of noise and linewidth on in vivo analysis of glutamate at 3 T. J Magn Reson (2020) 314:106732. 10.1016/j.jmr.2020.106732 32361510 PMC8485252

[108] Mescher M Merkle H Kirsch J Garwood M Gruetter R Simultaneous in vivo spectral editing and water suppression. NMR Biomed (1998) 11:266–72. 10.1002/(SICI)1099-1492(199810)11:6<266::AID-NBM530>3.0.CO;2-J 9802468

[109] Hurd R Sailasuta N Srinivasan R Vigneron DB Pelletier D Nelson SJ Measurement of brain glutamate using TE-averaged PRESS at 3T. Magn Reson Med (2004) 51:435–40. 10.1002/mrm.20007 15004781

[110] Schubert F Galliant J Seifert F Rinneberg H Glutamate concentrations in human brain using single voxel proton magnetic resonance spectroscopy at 3 Tesla. Neuroimage (2004) 21:1762–71. 10.1016/j.neuroimage.2003.11.014 15050596

[111] Choi CH Dimitrov IE Douglas D Patel A Kaiser LG Amezcua CA Improvement of resolution for brain coupled metabolites by optimized H-1 MRS at 7 T. NMR BioMed (2010) 23:1044–52. 10.1002/nbm.1529 20963800

[112] An L Li S Murdoch JB Araneta MF Johnson C Shen J Detection of glutamate, glutamine, and glutathione by radiofrequency suppression and echo time optimization at 7 Tesla. Magn Reson Med (2015) 73:451–8. 10.1002/mrm.25150 24585452

[113] Zhang Y Shen J Simultaneous quantification of glutamate and glutamine by J-modulated spectroscopy at 3 Tesla. Magn Reson Med (2016) 76:725–32. 10.1002/mrm.25922 PMC4788985 26361892

[114] Saleh MG Oeltzschner G Chan KL Puts NAJ Mikkelsen M Schär M Simultaneous edited MRS of GABA and glutathione. Neuroimage (2016) 142:576–82. 10.1016/j.neuroimage.2016.07.056 PMC5159266 27534734

[115] Oeltzschner G Saleh MG Rimbault D Mikkelsen M Chan KL Puts NAJ Advanced Hadamard-encoded editing of seven low-concentration brain metabolites: Principles of HERCULES. Neuroimage (2019) 185:181–90. 10.1016/j.neuroimage.2018.10.002 PMC6289748 30296560

[116] Medford N Critchley HD Conjoint activity of anterior insular and anterior cingulate cortex: awareness and response. Brain Struct Funct (2010) 214:535–49. 10.1007/s00429-010-0265-x PMC2886906 20512367

[117] Lee E Lee J Kim E Excitation/inhibition imbalance in animal models of autism spectrum disorders. Biol Psychiatry (2017) 81:838–47. 10.1016/j.biopsych.2016.05.011 27450033

[118] Sridharan D Levitin DJ Menon V A critical role for the right fronto-insular cortex in switching between central-executive and default-mode networks. Proc Natl Acad Sci U S A (2008) 105:12569–74. 10.1073/pnas.0800005105 PMC2527952 18723676

[119] Taylor KS Seminowicz DA Davis KD Two systems of resting state connectivity between the insula and cingulate cortex. Hum Brain Mapp (2009) 30:2731–45. 10.1002/hbm.20705 PMC6871122 19072897

[120] Horn DI Yu C Steiner J Buchmann J Kaufmann J Osoba A Glutamatergic and resting-state functional connectivity correlates of severity in major depression - the role of pregenual anterior cingulate cortex and anterior insula. Front Syst Neurosci (2010) 4:33. 10.3389/fnsys.2010.00033 20700385 PMC2914530

[121] Maclaren J Herbst M Speck O Zaitsev M Prospective motion correction in brain imaging: a review. Magn Reson Med (2013) 69:621–36. 10.1002/mrm.24314 22570274

[122] Evans CJ Puts NA Robson SE Boy F McGonigle DJ Sumner P Subtraction artifacts and frequency (mis-)alignment in J-difference GABA editing. J Magn Reson Imaging (2013) 38:970–5. 10.1002/jmri.23923 23188759

[123] An L Araneta MF Johnson C Shen J Effects of carrier frequency mismatch on frequency-selective spectral editing. MAGMA (2019) 32:237–46. 10.1007/s10334-018-0717-5 PMC6995993 30467687

[124] Geen H Freeman R Band-selective radiofrequency pulses J. Magn Reson (1991) 93:93–141. 10.1016/0022-2364(91)90034-Q

[125] McLean MA Busza AL Wald LL Simister RJ Barker GJ Williams SR In vivo GABA+ measurement at 1.5T using a PRESS-localized double quantum filter. Magn Reson Med (2002) 48:233–41. 10.1002/mrm.10208 12210931

[126] Choi C Coupland NJ Hanstock CC Ogilvie CJ Higgins AC Gheorghiu D Brain gamma-aminobutyric acid measurement by proton double-quantum filtering with selective J rewinding. Magn Reson Med (2005) 54:272–9. 10.1002/mrm.20563 16032672

[127] Choi IY Lee SP Merkle H Shen J In vivo detection of gray and white matter differences in GABA concentration in the human brain. Neuroimage (2006) 33:85–93. 10.1016/j.neuroimage.2006.06.016 16884929

[128] Simister RJ McLean MA Barker GJ Duncan JS Proton MR spectroscopy of metabolite concentrations in temporal lobe epilepsy and effect of temporal lobe resection. Epilepsy Res (2009) 83:168–76. 10.1016/j.eplepsyres.2008.11.006 19118980

[129] Coyle JT The GABA-glutamate connection in schizophrenia: which is the proximate cause? Biochem Pharmacol (2004) 68:1507–14. 10.1016/j.bcp.2004.07.034 15451393

[130] Cherlyn SY Woon PS Liu JJ Ong WY Tsai GC Sim K Genetic association studies of glutamate, GABA and related genes in schizophrenia and bipolar disorder: a decade of advance. Neurosci Biobehav Rev (2010) 34:958–77. 10.1016/j.neubiorev.2010.01.002 20060416

